# MCM6 promotes metastasis of hepatocellular carcinoma via MEK/ERK pathway and serves as a novel serum biomarker for early recurrence

**DOI:** 10.1186/s13046-017-0669-z

**Published:** 2018-01-22

**Authors:** Mingyu Liu, Qiaoting Hu, Mengxian Tu, Xinyi Wang, Zike Yang, Guoxiong Yang, Rongcheng Luo

**Affiliations:** 1 Cancer Center, Integrated Hospital of Traditional Chinese Medicine, Southern Medical University, Guangzhou, Guangdong 510315 China; 2 Cancer center, Southern Medical University, Guangzhou, Guangdong 510315 China; 3 Affiliated Cancer Hospital & Institute of Guangzhou Medical University, Guangzhou, Guangdong 510095 China; 4 Medical Imaging Center, Shenzhen Hospital of Southern Medical University, Shenzhen, Guangdong 518100 China

**Keywords:** Minichromosome maintenance complex component 6, MEK/ERK pathway, Metastasis, EMT, Survival

## Abstract

**Background:**

The high incidence of recurrence and metastasis of hepatocellular carcinoma (HCC) necessitate the discovery of new predictive biomarkers of invasion and prognosis. Minichromosome maintenance complex component 6 (MCM6), which has been reported to up-regulate in multiple malignancies, was considered to be a novel diagnoses biomarker in HCC. However, its functional contributions and prognostic value remain unclear.

**Methods:**

The expression of MCM6 was analyzed in 70 HCC tissues and 5 HCC cell lines by immunohistochemistry and real-time RT-PCR. The roles of MCM6 in HCC cell proliferation, migration and invasion were explored by CCK8, Wound healing and Transwell assays, respectively. Western blotting and Immunofluorescence staining were conducted to detect the protein expressions of ERK signaling pathway and EMT-related markers. To verify the above findings in vivo, we established subcutaneous xenograft tumor and orthotopic xenograft tumor models in nude mice. Finally, Enzyme-linked immunosorbent assay was used to evaluate the serum MCM6 level.

**Results:**

MCM6 was significantly up-regulated in HCC tissues. Increased MCM6 expression was associated with aggressive clinicopathological features and worse prognosis in HCC patients. These results were consistent with our analyses of The Cancer Genome Atlas database (TCGA). Furthermore, knockdown of MCM6 significantly decreased proliferative and migratory/invasive capability of HCC cells in vitro, as well as decreased tumor volume, weight and the number of pulmonary metastases in vivo. Mechanistic analyses indicated that MCM6 promoted EMT and activated MEK/ERK signaling. More importantly, serum MCM6 levels in HCC patients were significantly higher than those in cirrhosis and healthy controls (*P* < 0.0001), and allowed distinguishing early recurrence with high accuracy (AUC = 0.773).

**Conclusions:**

Our findings indicate that MCM6 predicts poor prognosis and promotes metastasis in HCC. Postoperative serum MCM6 level could be valuable to detect preclinical early recurrence, indicative of a need for more careful surveillance and aggressive therapeutic intervention.

**Electronic supplementary material:**

The online version of this article (10.1186/s13046-017-0669-z) contains supplementary material, which is available to authorized users.

## Background

Hepatocellular carcinoma (HCC) is one of the most prevalent malignant tumors worldwide. Although surgical techniques and adjuvant therapy have improved, long-term survival remains low due to tumor recurrence and metastasis [1]. Early recurrence is defined as HCC that recurs within 1 year after resection, and it is most closely related to cancer metastasis spread and is the leading cause of early death in patients with HCC after liver resection [2]. Therefore, it is critical to understand the molecular mechanism associated with HCC invasion and metastasis and identify the predictive biomarkers of HCC recurrence and prognosis to help monitor disease progression and guide diagnosis and treatment.

The Mini-chromosome maintenance protein (MCM) family is a recently reported gene family that was identified in the control of eukaryotic genome replication. In the DNA replication licensing process, the MCM2–7 complex primes chromatin for DNA replication by binding origins of DNA replication during the late M to early G1 phase of the cell cycle [3]. Also, it is involved in the formation of replication forks and in the recruitment of other DNA-replication-related proteins. The MCM complex is a replicative helicase that is essential for DNA replication initiation and elongation in eukaryotic cells [4].

The MCM protein family plays an important role in genome duplication of proliferating cells. It had been reported that abnormal expression of MCMs contributes to tumorigenesis. For example, a significant rise in the MCM5 level was observed in cervical epithelium squamous cancer [5]. MCM7 expression may predict poor postoperative prognosis for HCC and promote cancer progression through cyclin-D1-dependent signaling [6, 7]. Altered Mcm5, Mcm2 and Mcm7 were found in malignant cells in the lung, kidney and prostate [8–11]. Furthermore, a rise of MCM6 has been shown to correspond with a high risk of recurrence in meningioma [12], suggesting that the MCM proteins are promising prognostic markers for monitoring various cancers.

MCM6 is one of six members of the Mini-chromosome maintenance family. Previous studies verified the important role of MCM6 in cell proliferation and indicated its potential role in prompting tumor progression. MCM6 is up-regulated in various types of tumors [13–15]. It has been reported that a high serum MCM6 level is a diagnostic biomarker for HCC [16]. However, little is known about its relation to cancer survival or its prognostic significance in HCC. The previous studies give rise to the hypothesis that MCM6 plays an important role in HCC. In the present study, we performed a series of experiments to explore its molecular function in HCC cells and evaluate the prognostic value of both the tissue and plasma MCM6 expression level.

## Methods

### Tissue and serum specimens

Tumor tissue specimens, including tumor and matched adjacent non-tumor tissues, were obtained from 70 patients who had undergone curative liver resection. Detailed clinical pathological parameters were listed in Table 1. Thirty normal hepatic tissues were obtained from patients suffering from benign hepatic lesions who underwent resection. The serum samples, including 34 healthy people, 32 patients with cirrhosis, and 31 patients with HCC (obtained by collecting venous blood at the time of the primary diagnosis before operation and 8 days after surgical resection) were used in this study for measuring the serological level of MCM6 by ELISA. Serum samples were frozen and stored at −80 °C until measurement. All the selected HCC cases for serological examination had received curative hepatectomy and been provided with a uniform follow-up to the cohort. Curative hepatectomy was defined to involve (1) complete removal of all nodules with the resection margin greater than 10 mm, (2) the absence of invasion of the main trunk and first-order branches of the portal vein, common hepatic duct and its first-order branches or main trunk of the hepatic vein and inferior vena cava (3) the absence of intra- or extra-hepatic metastasis, and (4) the absence of residual tumor or portal tumor thromboses on postoperative imaging. In this study, all specimens were obtained and used under protocols approved by the Integrated Hospital of Traditional Chinese Medicine of Southern Medical University Office for Protection of Human Subjects.

**Table 1:** Correlation of MCM6 protein expression with clinicopathological parameters

Characteristics	n	MCM6		*P* value
		negative	positive	
Age (years)				
≤ 50	36	7 (19.4%)	29 (80.6%)	0.111
> 50	34	12 (35.3%)	22 (64.7%)	
Serum AFP (μg/l)				
≤ 20	18	11 (61.1%)	7 (38.9%)	**0.000**
> 20	52	8 (15.4%)	44 (84.6%)	
HBsAg				
Negative	8	5 (62.5%)	3 (37.5%)	**0.035**
Positive	60	15 (25.0%)	45 (75.0%)	
GGT (U/I)				
≤ 50	28	8 (28.6%)	20 (71.4%)	0.518
> 50	42	11 (26.2%)	31 (73.8%)	
Child-Pugh score				
A	66	17 (25.8%)	49 (74.2%)	0.603
B	3	1 (33.3%)	2 (66.7%)	
Tumor capsule				
No/incomplete	61	16 (26.2%)	45 (73.8%)	0.463
Complete	9	3 (33.3%)	6 (66.7%)	
Tumor differentiation				
I-II	63	16 (25.4%)	47 (74.6%)	0.399
III-IV	5	2 (40.0%)	3 (60.0%)	
Vascular invasion				
No	63	19 (30.2%)	44 (69.8%)	0.097
Yes	7	0 (0%)	7 (100%)	
Liver cirrhosis				
No	31	14 (45.2%)	17 (54.8%)	**0.003**
Yes	39	5 (12.8%)	34 (87.2%)	
Tumor size (cm)				
≤ 5	34	11 (32.4%)	23 (67.6%)	0.247
> 5	36	8 (22.2%)	28 (77.8%)	
Tumor number				
Solitary	49	18 (36.7%)	31 (63.3%)	**0.004**
Multiple	21	1 (4.8%)	20 (95.2%)	
Early recurrence				
No	36	15 (41.7%)	21 (58.3%)	**0.002**
Yes	33	3 (9.1%)	30 (90.9%)	

(*P* < 0.05) had been marked in boldface

### Patients in the TCGA database

MCM6 expression data based on RNA-Seq were extracted from The Cancer Genome Atlas (TCGA) database for 291 patients with HCC. Patients were divided into low and high expression groups, and the prognostic values of MCM6 for overall survival, cumulative recurrence, and early-recurrence were assessed.

### Cell culture

HCC cell lines (HCC-LM3, SMMC7721, Huh7 and PLC/PRF/5) were obtained from the Cancer Research Institute of Southern Medical University in Guangzhou, China. HepG2 cells were purchased from the Chinese Academy of Sciences Cell Bank (Shanghai, China). All of the cells were routinely maintained in high-glucose DMEM (Life Technologies, Grand Island, NY, USA) supplemented with 10% fetal bovine serum (Life Technologies) at 37 °C with 5% CO_2_.

### Lentivirus production and infection

Lentiviral particles expressing shRNA against MCM6 or control sequence were constructed by Cyagen Bioscience Inc. (Guangzhou, China). LM3 and SMMC-7721 cells were transfected with lentiviral vectors and selected with 750 mg/mL G418 (Gibco). MCM6 expression was confirmed by qPCR, and the levels of MCM6 protein were measured using western blotting.

### RNA isolation, reverse transcription, and real-time RT-PCR

Total RNA was isolated from cells using the E.Z.N.A. Total RNA Kit I (Cat. No 66834–02 Omega). Reverse transcription was performed using the PrimeScript RT Reagent Kit (TaKaRa Biotech, Dalian, China). qRT-PCR reactions were performed using a Bio-Rad system (Bio-Rad Labs, Hercules, CA) with SYBR Green PCR Master Mix (TaKaRa Biotech). The cycling conditions were denaturation at 95 °C for 15 s, with annealing at 60 °C for 15 s and fluorescence collection at 72 °C for 10 s. The 2 − ∆∆Ct method was used for relative quantification and statistical analysis. Independent experiments were done in triplicate. The primers used in this study are listed in the Additional file 1: Table S1.

### Immunohistochemistry assay

Immunohistochemical staining of tissue was performed according to the manufacturer’s instructions (DAKO, Glostrup, Denmark) on formalin-fixed, paraffin-embedded tissue sections that had been cut to 4-μm thickness. The specimens were dried at 62 °C for 2 h and then dewaxed in xylene and rehydrated using graded alcohols, followed by incubation in 3% hydrogen peroxide for 15 min to exhaust the endogenous peroxidase activity. The antigens were then retrieved in Tris-EDTA (pH 9.0)/ 0.01 M sodium citrate buffer (pH 6.0) using a microwave oven for 25 min, followed by blocking with 10% goat serum for 30 min to prevent non-specific staining. Primary antibodies were incubated overnight at 4 °C in a humidified chamber, followed by HRP-labeled anti-mouse/rabbit secondary antibody (DAKO) incubation for 1 h at room temperature. Antibody binding was detected by DAB, and the reaction was stopped by immersion in distilled water once the brown color appeared. Finally, specimens were counter-stained with hematoxylin for 3 min. Two different pathologists who specialized in liver cancer evaluated the IHC results without knowledge of the clinical data. Both the extent and intensity of immunostaining were taken into consideration when analyzing the data. The intensity of staining was scored from 0 to 3, and the extent of staining was scored from 0% to 100%. Final quantitation of staining was obtained by multiplying the two scores. The protein expression was classified as high expression if the score was higher than 1.5 and low expression if the score was 1.5 or less. The antibodies are listed below: MCM6 (1:4000; Abcam, Cambridge, UK), Ki-67 (1:200, ZSGB-BIO, China), Vimentin (1:100, ZSGB-BIO, China) and E-cadherin (1:100, ZSGB-BIO, China).

### Immunofluorescence staining

Cells grown on coverslips were rinsed with phosphate-buffered saline (PBS) and fixed with cold 4% paraformaldehyde for 5 min at RT. Subsequently, the cells were blocked with Triton X-100 at a concentration of 0.2% for 30 min. Cells were then blocked for 1 h with 5% BSA and washed for 30 min, followed by incubation with primary monoclonal antibodies against MCM6 (1:500; Abcam, Cambridge, UK). Vimentin (1:100, ZSGB-BIO, China) and E-cadherin (1:50, ZSGB-BIO, China) overnight at 4 °C. The next day, the coverslips were incubated for 1 h in a dark room with Alexa Fluor 564 goat anti-rabbit IgG and Alexa Fluor 488 goat anti-mouse IgG (1:100 dilution; Bioworld Technology, Inc). Furthermore, the coverslips were stained with DAPI for 5 min at 4 °C. Finally, an LSM80 fluorescent microscope (ZESS, Germany) was used to observe the expression in cells.

### Western blot

Total proteins were extracted with RIPA lysis buffer supplemented with protease inhibitors, separated by SDS-PAGE and transferred to a PVDF membrane (Millipore, Billerica, MA). The membrane was blocked with 5% non-fat milk at room temperature for 1 h and incubated with the appropriate antibody. Protein bands were visualized by the chemiluminescent HRP detection system (Millipore, Billerica, MA). The antibodies are listed below: MCM6 (1:2000, Proteintech, Chicago, IL), E-cadherin (1:1000, CST, Boston, MA), Vimentin (1:1000, CST, Boston, MA), GAPDH (1:5000, Ray antibody, China), ZO1 (1:1000, CST, Boston, MA), Fibronectin (1:1000, Wanleibio, China), p-ERK1/2 (1:300, Wanleibio, China), ERK1/2(1:500, Wanleibio, China), p-MEK1/2 (1:500, ABclonal, China), and MEK1/2 (1:1000, Wanleibio, China).

### Cell viability assay

Cell viability was analyzed using the Cell Counting Kit-8 (CCK-8) (Dojindo Molecular Technologies Inc. Shanghai, China). Cells at a density of 2 × 10^3^/well were seeded into 96-well plates and cultured in 100 μL of DMEM containing 10% FBS for 4 days. Ten microliters of CCK8 solution was added to each plate, and the cells were incubated for 3 h at 37 °C. The absorbance value (OD) of each well was measured at 450 nm.

### EDU incorporation assay

For the EdU incorporation assay, proliferating cells were examined using the Cell-Light EdU Apollo 567 in vitro Imaging Kit (RiboBio, Guangzhou, China) according to the manufacturer’s protocol. Cells at a density of 5 × 10^3^/well were seeded into 96-well plates and incubated with 10 mM EdU for 2 h, followed by fixation with 4% paraformaldehyde, permeabilization in 0.2% Triton X-100 and staining with Apollo fluorescent dyes. Next, 50 μL/well DAPI was used to stain the cell nuclei for 10 min. The number of EdU-positive cells was counted under a fluorescent microscope in five random fields.

### Wound-healing assay

For the wound-healing assay, cells were grown to confluence in a six-well plate. Artificial wound tracks were created by scraping the confluent cell monolayers with a pipette tip. The cells were fed with serum-free medium. The ability of the cells to migrate into the wound area was assessed every 24 h after scratching.

### Colony formation assay

Cells were plated in 6-well culture plates at 100 cells/well. After incubation for 2 weeks at 37 °C, the cells were washed twice with PBS and stained with 0.1% crystal violet solution. The number of colonies containing ≥50 cells was counted under a microscope. The colony formation efficiency was calculated as (number of colonies/number of cells inoculated) × 100%.

### Cell migration and invasive assays

Cell motility was assessed by cell migration and invasion assays using transwell chambers with or without Matrigel (BD, Biosciences, CA). Approximately 6 × 10^4^ cells in medium without FBS were seeded on transwell chambers with or without Matrigel and incubated at 37 °C for 15 h. Medium containing 10% FBS was put in the lower chamber. The invasive cells attached to the lower surface of the membrane insert were fixed, stained using Giemsa (Jiancheng, Jiangsu, China) and quantified.

### Enzyme-linked immunosorbent assay (ELISA)

The serologic level of MCM6 was detected using the human MCM6 ELISA kit (Cusabio, Wuhan, CN) according to the manufacturer’s instructions. One hundred microliters of serum samples and standard proteins was added to the appropriate wells and incubated at 37 °C for 2 h. After being washed three times with washing buffer, HRP-conjugated secondary antibody (100 μL/well) was added to the cells, which were incubated at 37 °C for 1 h. The plate was washed three times, and the cells were then incubated with DAB solution for 15–30 min at 37 °C. Then, 50 μL of Stop Solution was added to each well, the optical density at 450 nm was detected, and the concentration of MCM6 was calculated from the standard curve.

### Animal studies

Both subcutaneous and orthotopic models were used for animal studies. A total of 2 × 10^6^ SMMC7721 cells transfected with MCM6 shRNA, lentiviral vectors and negative control (scramble) vector in 0.1 ml PBS medium was used in both the subcutaneous model and orthotopic model. The cells were injected into the left leg of 4–6-week-old male BALB/c nude mice. The subcutaneous tumor size was calculated and recorded every 3 days with a Vernier caliper using the following equation: tumor volume (mm^3^) = (L × W^2^)/2, where L = tumor long axis and W = short axis. The measurements were repeated three times. The mice were maintained in a barrier facility on HEPA-filtered racks and fed an autoclaved rodent diet. After 27 days, the mice were killed and the tumor tissues were surgically excised, weighed and stained with hematoxylin and eosin (H&E). For the orthotopic model, the cell suspension was injected into the left hepatic lobe of 4-week-old nude mice with a micro-syringe. Transplanted cells were allowed to grow for up to 6 weeks, when the mice were sacrificed. The livers and lungs were dissected, fixed, and paraffin-embedded. All animal handling and procedures were approved by the Animal Care and Use Committee of Southern Medical University.

### Statistical analysis

All statistical analyses were performed with SPSS statistical software (version 21.0; SPSS, IBM, Armonk, NY). Survival curves were constructed using the Kaplan–Meier method and analyzed by the log-rank test. Significant prognostic factors found by univariate analysis were entered into a multivariate analysis using the Cox proportional hazard regression model. The Pearson’s chi-square test was used to analyze the association of MCM6 expression with various clinicopathologic characteristics. A paired Student’s t test or χ2 tests was used to compare the values between subgroups. Receiver operating characteristic (ROC) curve analysis was used to determine the predictive value of the parameters, and the differences in the area under the curve (AUC) were detected. R project for Statistical Computing (R version 3.4.3) was used to calculate Harrell C-index. Data were expressed as the mean ± SD of at least three sample replicates. A value of *P* < 0.05 was statistically significant, and single, double, and triple asterisks indicate statistical significance of * *P* < 0.05, ** *P* < 0.01 and *** *P* < 0.001.

## Results

### MCM6 expression is significantly up-regulated in HCC tissues and correlates with poor prognosis and high recurrence rates in HCC patients

**Fig. 1:**
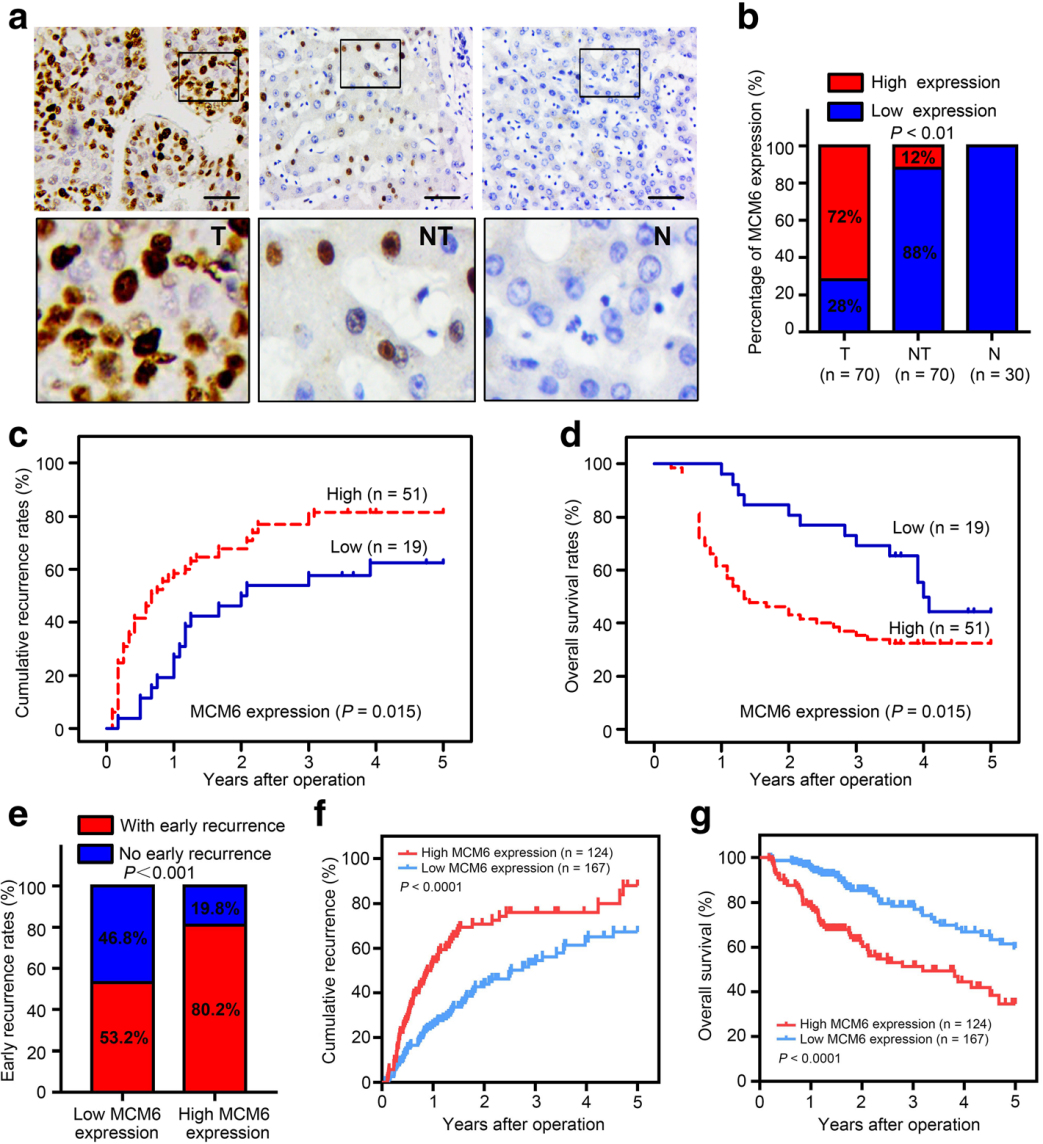
MCM6 expression is significantly up-regulated in HCC and indicates poor prognosis. **a** Representative IHC images of MCM6 expression in 30 normal hepatic tissues and 70 paired HCC and adjacent non-tumor tissues. The upper left two panels represent paired HCC and non-tumor tissues and reveal high MCM6 expression in HCC tissues and low expression in adjacent non-tumor tissues. The upper right panel represents normal hepatic tissues and reveals no MCM6 expression. Scale bars, 50 μm. Lower panels represent magnified pictures of the boxed area in the corresponding upper panels. **b** MCM6 expression levels were compared among HCC, adjacent non-tumor and normal hepatic tissue specimens. ** *P* < 0.01. **c**-**d** Prognostic significance assessed by Kaplan–Meier survival estimates and log rank tests. Comparison of the time to recurrence (TTR) and overall survival (OS) by MCM6. **e** MCM6 was correlated with early recurrence in HCC patients based on TCGA datasets. **f**-**g** TCGA clinical data showing TTR and OS curves for HCC of MCM6 expression

To evaluate the expression of MCM6 in HCC tissues, we first conducted immunohistochemistry assay in 70 HCC specimens (tumor and matched adjacent non-tumorous tissues) and 30 normal hepatic tissue specimens. We observed that MCM6 was primarily localized in the nucleus. The results revealed that MCM6 expression in HCC tissues was markedly higher than the adjacent non-tumor liver tissue and normal liver tissue (Fig. 1a). We detected high expression of MCM6 in 50/70 (71.4%) HCC tissues, compared with only 8/70 (11.4%) adjacent non-tumor tissues and no staining in normal tissues (*P* < 0.01; Fig. 1b).

Second, to verify the functions of MCM6, we tested the correlation of MCM6 expression status in 70 HCC specimens with 12 widely recognized clinicopathological parameters. Statistical analysis indicated that high MCM6 expression was associated with hepatitis B infection, liver cirrhosis, multiple tumor nodules, high serum AFP and early recurrence (Table 1). Furthermore, univariate analysis revealed that tumor size, GGT level, vascular invasion, early recurrence and MCM6 expression were unfavorable predictors for TTR and OS (Table 2). To assess the prognostic significance of MCM6, we do the Kaplan–Meier survival analysis. The 1-, 3- and 5-year recurrence rates in the MCM6-positive group were significantly higher than in the MCM6-negative group (58.0%, 80.1% and 81.5% versus 19.2%, 55.1% and 61.9%, respectively; Fig. 1c). Similarly, the 1-, 3- and 5-year OS rates in the MCM6-positive group were significantly lower than in the MCM6-negative group (61.2%, 38.3% and 32.4% versus 95.1%, 70.7% and 45.1%, respectively; Fig. 1d). Further multivariate analysis demonstrated that MCM6 was an independent prognostic factor for both the OS (*P* < 0.05) and TTR (*P* < 0.05) of HCC patients (Table 3).

**Table 2:** Univariate analysis of factors associated with survival and recurrence

Variables	OS		TTR	
	Hazard ratio (95% CI)	*P* value	Hazard ratio (95% CI)	*P* value
Age (years)	0.632 (0.349–1.142)	0.128	0.722 (0.425–1.229)	0.231
AFP (μg/l)	0.335 (0.141–0.796)	**0.013**	0.529 (0.272–1.028)	0.060
HBsAg	2.451 (0.758–7.925)	0.134	2.227 (0.802–6.186)	0.124
GGT (U/I)	1.991 (1.056–3.754)	**0.033**	1.869 (1.067–3.274)	**0.029**
Child-Pugh score	1.437 (0.347–5.940)	0.617	1.160 (0.282–4.769)	0.837
Tumor capsule	0.902 (0.381–2.132)	0.814	1.266 (0.542–2.956)	0.586
Tumor differentiation	1.778 (0.633–4.994)	0.275	1.415 (0.510–3.926)	0.505
Vascular invasion	2.433 (1.015–5.833)	**0.043**	2.563 (1.141–5.760)	**0.023**
Liver-cirrhosis	1.357 (0.915–3.112)	**0.048**	1.845 (1.055–3.228)	**0.028**
Tumor size (cm)	1.893 (1.322–3.473)	**0.039**	1.856 (1.077–3.199)	**0.026**
Tumor number	1.438 (0.812–2.203)	0.210	1.256 (0.860–1.903)	0.392
Early recurrence	1.047 (0.418–2.525)	**0.005**	1.709 (0.583–1.859)	**0.023**
MCM6 expression	2.001 (1.043–2.187)	**0.021**	1.819 (1.292–2.561)	**0.021**

(*P* < 0.05) had been marked in boldface

**Table 3:** Multivariate analysis of factors associated with survival and recurrence

Variables	OS		TTR	
	Hazard ratio (95% CI)	*P* value	Hazard ratio (95% CI)	*P* value
Vascular invasion	1.230 (0.484–3.128)	**0.046**	1.311 (0.548–3.133)	0.072
Liver cirrhosis	2.687 (0.915–3.112)	0.094	1.845 (1.055–3.228)	**0.032**
Tumor size (cm)	1.792 (1.031–2.672)	**0.041**	1.731 (1.068–3.034)	**0.031**
Early recurrence	1.130 (0.499–1.826)	**0.033**	1.819 (1.067–3.274)	0.069
MCM6 expression	1.420 (0.191–0.924)	**0.031**	1.481 (0.244–0.950)	**0.035**

(*P* < 0.05) had been marked in boldface

In addition, to confirmed the prognostic value of MCM6, we applied MCM6 to 291 HCC samples with RNA-sequencing data in the Cancer Genome Atlas project (TCGA). Based on the relative expression of MCM6, 124 and 167 patients were classified into high- and low- expression groups, respectively. We performed systematic Kaplan-Meier survival and clinicopathological features analysis of the whole mRNA transcriptomics and found that high MCM6 mRNA expression was significantly correlated with early recurrence (recurrence rates 80.2% vs. 53.2%) (Fig. 1e) and predicted poor survival and high recurrence risk (Fig. 1f-g).

### Knockdown of MCM6 suppresses HCC cell migration, invasion and proliferation in vitro

**Fig. 2:**
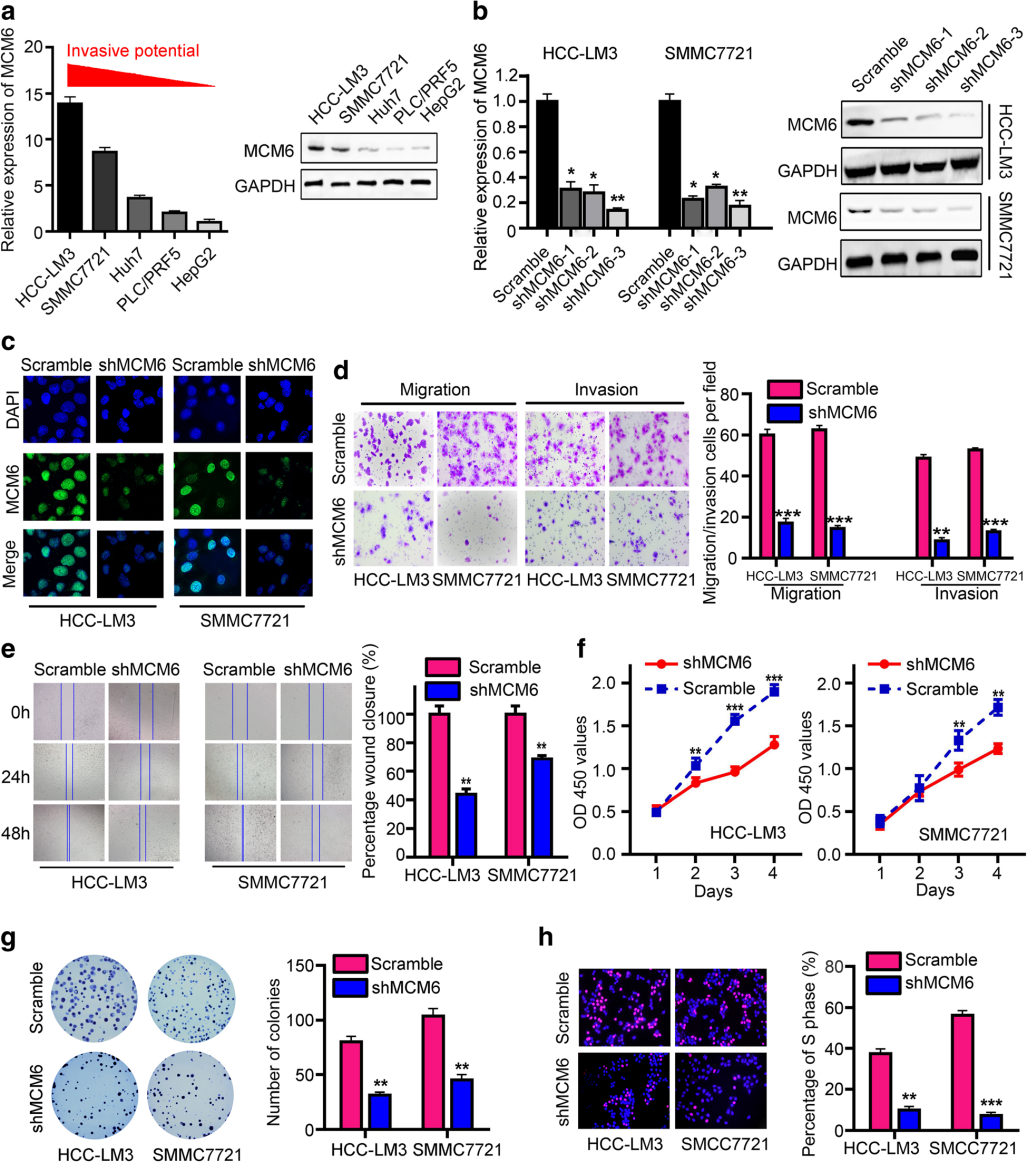
MCM6 enhances HCC cell migration, invasion and proliferation properties in vitro*.* **a** Expression of MCM6 in hepatocellular carcinoma cell lines analyzed by qRT-PCR and western blotting. **b**-**c** Expression levels of MCM6 were measured by qRT-PCR, western blot and immunofluorescence in HCC cells treated with LV-shMCM6 or LV-Scramble. **d** Representative images and quantification of the number of cells that migrated or invaded in the indicated stable cell lines. Experiments were repeated three times with similar results, and error bars represent the mean ± SEM, ***P < 0.001. **e** Wound-healing assays were employed to detect the migration capacity of MCM6-altered cells. The in vitro proliferation function of MCM6 was measured by the CCK8 assay (**f**), colony-forming assay (**g**) and Edu assays (**h**)

In vitro experiments suggested that MCM6 expression in high-metastasis potential cell lines, such as HCC-LM3 and SMMC7721, was higher than in the low-metastasis potential cell lines PLC/PRF5 and HepG2 (Fig. 2a). These data indicated that MCM6 might regulate the invasion and metastasis of HCC cells. This speculation was confirmed by cytological experiments. To understand the function of MCM6 in HCC cells, we designed three short hairpin RNAs (shRNA1, shRNA2, and shRNA3) to silence MCM6 expression. More than fourfold decrease in MCM6 expression was observed in SMMC7721 and HCC-LM3 cells treated with shRNA compared with the control group by qRT-PCR (Student’s t-test, with *P* < 0.05 for both) and western blotting; shRNA3 was the most effective shRNA and was chosen for further study (Fig. 2b-c). The wound-healing and Matrigel-coated (for invasion) or –uncoated (for migration) Transwell assays showed that HCC invasion and metastasis were effectively inhibited by MCM6 knockdown. The results showed that SMMC7721-scramble and HCC-LM3-scramble cells had a faster wound closure rate and more invasive properties than shMCM6 cells (Fig. 2d-e). These results uncovered the role of MCM6 in promoting HCC progression in vitro.

Subsequently, we examined the effect of MCM6 expression on HCC cells growth in vitro. Using CCK-8 assay (Fig. 2f), colony formation (Fig. 2g), and Edu incorporation assays (Fig. 2h), we found that knockdown MCM6 markedly suppressed cell proliferation in SMMC7721 and HCC-LM3 cells.

### MEK/ERK signaling activation is critical for MCM6-induced EMT

**Fig. 3:**
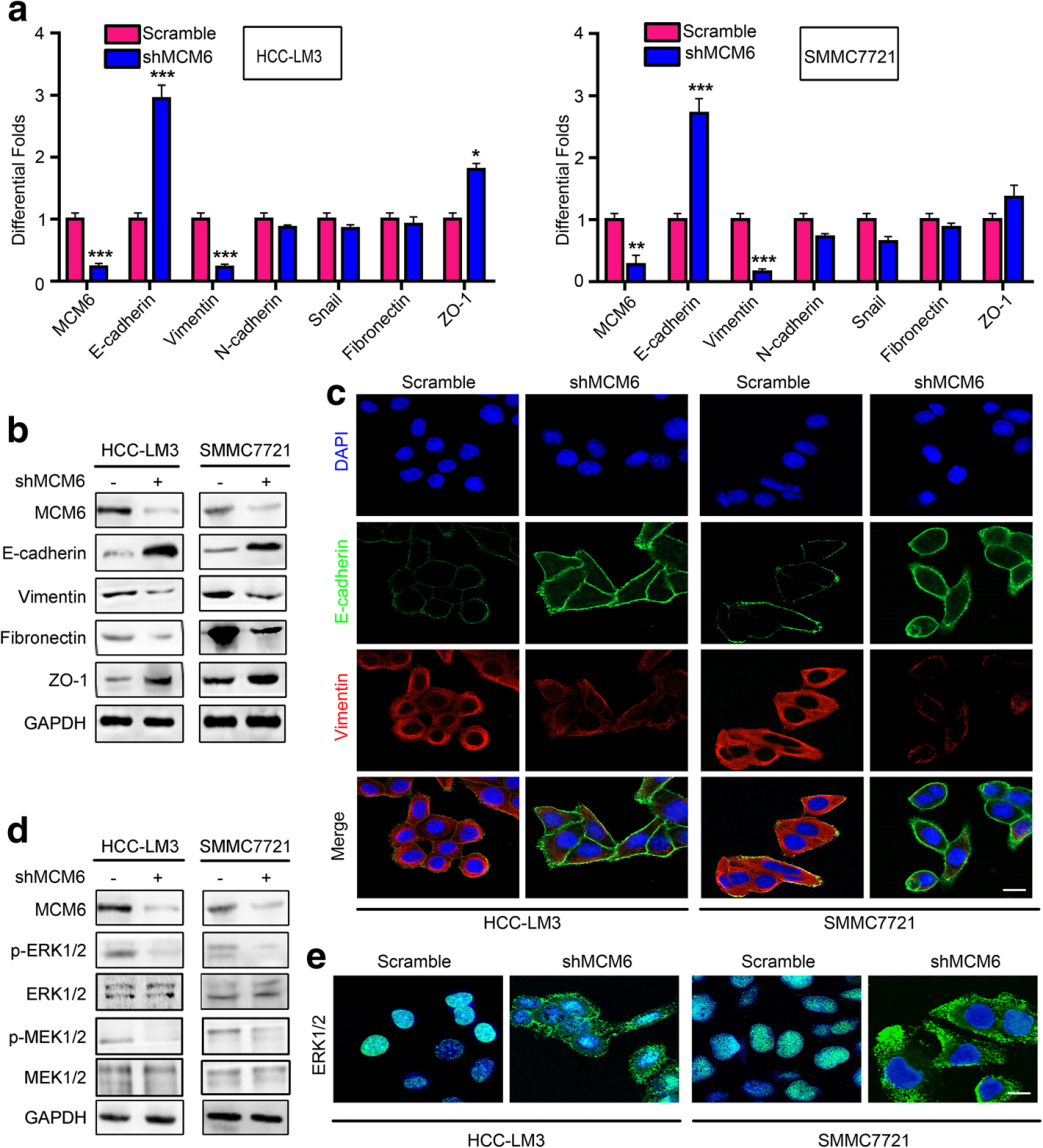
MCM6 promotes EMT through activating MEK/ERK signaling. **a**-**b** Analysis of the mRNA and protein levels of EMT-associated genes shows that MCM6 expression is inversely correlated with E-cadherin but positively correlated with vimentin expression. The other EMT markers were not obviously influenced by the change in MCM6. **c** Representative IF images of E-cadherin and vimentin expression in the cells treated with LV-shMCM6 or LV-Scramble. Scale bars: 20 μm. **d** Expression of MCM6, p-ERK, p-MEK, ERK, and MEK was detected by western blot. **e** Immunofluorescence confirmed that MCM6 participates in the nuclear expression of ERK. Scale bars, 20 μm

Invasion and metastasis are responsible for the unsatisfactory long-term survival. Emerging evidence suggested that EMT endows epithelial cells with their migratory and invasive capacity and is involved in many cancer metastasis processes, including HCC [17, 18]. To explore the relationship between MCM6 and the epithelial–mesenchymal transition (EMT), we performed real-time PCR to assess EMT markers in MCM6 knockdown cells. We found that the suppression of MCM6 resulted in higher E-cadherin and ZO-1 levels, accompanied by decreased levels of the mesenchymal marker vimentin, but did not significantly influence the other EMT markers (Fig. 3a). Western blot and immunofluorescence staining also confirmed that MCM6 expression inversely correlated with E-cadherin but positively correlated with vimentin expression (Fig. 3b and c).

Next, we sought to determine the signaling mechanisms involved in MCM6-mediated EMT. The extracellular signal-regulated kinase 1/2 (ERK, also known as p42/44 mitogen-activated protein kinase MAPK) signaling pathway is one of the most intensively studied signaling mechanisms that regulates migration, invasion, and EMT in cancer [19–21]. We found that MCM6 affected activation of ERK signaling. We analyzed the activation status of ERK and the upstream gene MEK1/2 in cells with stable MCM6 knockdown. The data showed that MCM6 increased the levels of phosphorylated MEK1/2 (p-MEK1/2) and ERK1/2 (p-ERK1/2), but the total MEK and ERK had similar expression levels in all experimental conditions (Fig. 3d). Meanwhile, we used immunofluorescence assays to detect the localization and expression of ERK protein. It is widely known that ERK shuttling between the nucleus and cytoplasm provides a switch-like transition effect in the activation of the ERK signal, and active nuclear ERK1/2 levels were assessed by immunofluorescence. Consistent with the western blot data, the confocal photos revealed that the activation of ERK1/2 was strongly suppressed when MCM6 was knocked down (Fig. 3e). Taken together, these data suggest that MCM6 promotes EMT, at least in part, through the upregulation of MEK/ERK signaling pathways.

### MCM6 augments tumor-initiating and metastatic potential in vivo

**Fig. 4:**
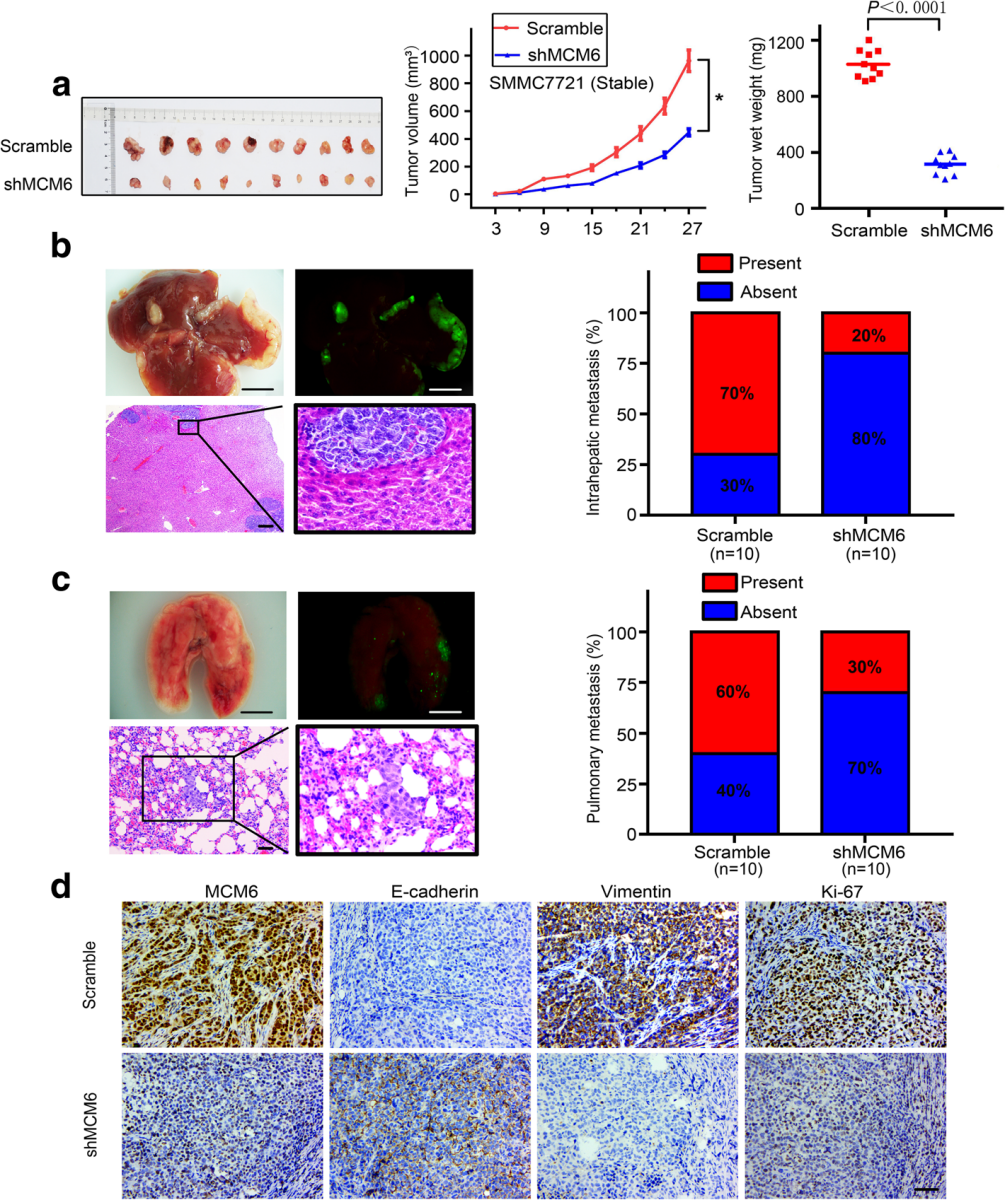
MCM6 augments tumor proliferation and metastatic potential in vivo. **a** Xenografts in nude mice were established by subcutaneous injection of SMMC7721-shMCM6 and control cells. **b** and **c** Representative pictures and external whole-body fluorescence images of intrahepatic and lung metastasis, which were obtained 6 weeks after orthotopic liver injection, respectively. *P < 0.05 based on Student’s t test. Scale bar: 5 mm (top), 100 μm (bottom). **d** Representative IHC images of MCM6, as well as Ki67 and EMT-associated genes in subcutaneous tumors of mice injected with SMMC7721-shMCM6 and control cells. Scale bars: 50 μm

To further investigate the effects of MCM6 in vivo, we established subcutaneous xenograft tumor and orthotopic xenograft tumor models in nude mice. After 4 weeks, SMMC7721-Scramble cell-derived tumors at the subcutaneous implantation sites were larger and grew more rapidly than SMMC7721-shMCM6 cell-derived tumors. (Fig. 4a). Consistently, the liver orthotopic xenograft tumor also showed that MCM6 knockdown had imposed significant regulatory effects on HCC metastasis. The intrahepatic and pulmonary metastasis rates in mice with tumors derived from SMMC7721-shMCM6 cells were significantly lower than the corresponding control group (Fig. 4b and c). Moreover, the effect of MCM6 on E-cadherin and vimentin was also observed by IHC in the xenograft specimens (Fig. 4d). We found that MCM6 expression inversely correlated with E-cadherin but positively correlated with vimentin and Ki-67 expression. Taking these results together, our studies demonstrated that MCM6 could promote HCC growth and metastasis in vivo*.*

### Elevated serum MCM6 lever is correlated with increased risk of early recurrence after curative hepatectomy

**Fig. 5:**
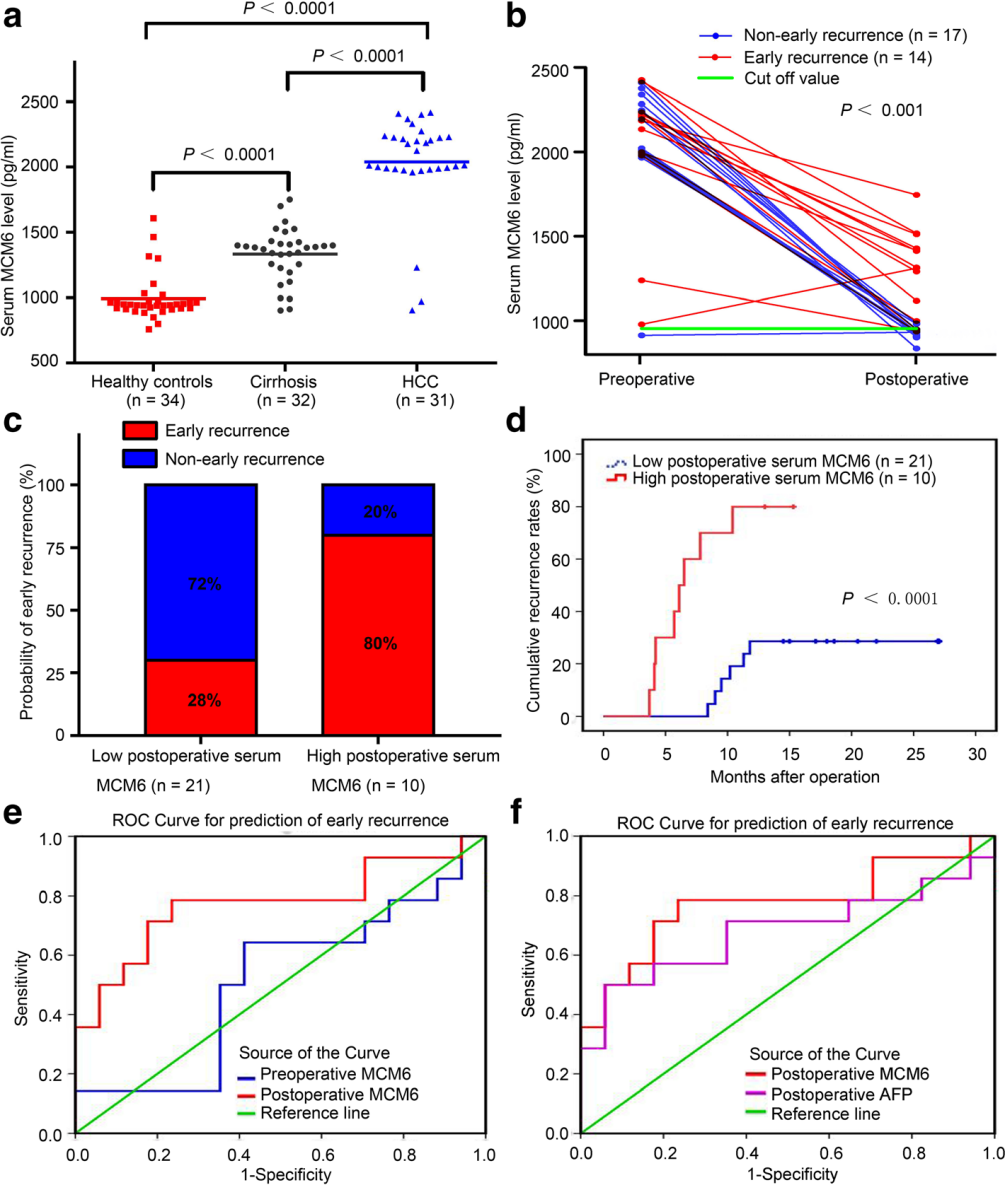
Elevated perioperative plasma MCM6 correlates significantly with increased risk of early recurrence after curative hepatectomy. **a** Compared with healthy control individuals and cirrhotic patients, serum MCM6 was significantly higher in patients with HCC (*P* < 0.0001). **b** In the non-early recurrence subgroup, plasma MCM6 levels decreased dramatically after operation and some were even reduced to the normal range in a certain time. **c** The high serum MCM6 subgroup was more likely to suffer from early recurrence than the low MCM6 subgroup after surgical resection (80% versus 28%). **d** A high serum MCM6 level correlated significantly with cumulative recurrence after curative hepatectomy (*P* < 0.0001). **e**-**f** ROC curve by serum biomarkers indicated that postoperative serum MCM6 has a significant predictive ability of HCC early recurrence: AUC ^postoperative MCM6^ > AUC ^preoperative MCM6^(0.773 versus 0.513), and AUC ^postoperative MCM6^ > AUC ^postoperative AFP^ (0.773 versus 0.681). ROC, receiver operating characteristic. AUC, area under the curve

Previous studies demonstrated that the MCM6 levels in plasma had diagnostic value for HCC [16]. Additionally, our earlier results revealed that the elevated expression of MCM6 in tissues and serum is correlated with early recurrence (Table 1) (Additional file 1: Table S2). Therefore, to further explore its potential value in early recurrence non-invasive serologic detection, we examined MCM6 expression in patient serum. Firstly, when discerning HCC patients from cirrhosis and healthy controls, serum MCM6 had significant sensitivity (*P* < 0.0001, Fig. 5a). Moreover, the data obviously revealed that the serum MCM6 concentration from HCC patients who received curative hepatectomy dropped sharply compared with the pre-treatment level (2039.17 ± 206.02 pg/mL preoperative versus 1077.51 ± 234.31 pg/mL postoperative, *P* < 0.05). In addition, in the non-early recurrence subgroup, plasma MCM6 levels declined more dramatically after operation and some were even reduced to the normal range over a certain time (Fig. 5b).

To analyze the value of serum MCM6 in predicting the prognosis of HCC, we prepared the receiver operating characteristic (ROC) curve for serum MCM6 expression according to early recurrence. The cut off value was determined using receiver operating characteristic (ROC) curves and the Youden’s Index. (Youden’s index = sensitivity + specificity-1). According to Youden’s index, optimal sensitivity (78.6%) and specificity (76.5%) were seen at a cut-off of ≥941.635 pg/ml to distinguish the non-early recurrence group from the early recurrence group. To estimate the value of the dynamic changes in serum MCM6 in the early recurrence of HCC after liver resection, we analyzed the differences in the recurrence rate of the two groups. Early-recurrence was observed in 8 of 10 (80%) patients with high postoperative MCM6 levels (≥ 941.635 pg/ml), whereas only 6 of 21 (28%) patients with low postoperative MCM6 levels (< 941.635 pg/ml) had early-recurrence (Fig. 5c). Additionally, a high postoperative MCM6 level correlated significantly with cumulative recurrence after curative hepatectomy (Fig. 5d).

Next, the predictive performance was compared between serum preoperative MCM6 and postoperative MCM6 (Fig. 5e). Our data showed that after curative hepatectomy, the area under the ROC curve (AUROC) for MCM6 was 0.773 (95% confidence interval [CI], 0.594–0.953). The postoperative serum MCM6 was more informative than the preoperative level in predicting early recurrence in HCC. Furthermore, the ROC analysis also revealed that postoperative MCM6 is superior to postoperative AFP in differentiating early recurrence. (Fig. 5f). The AUC of postoperative MCM6 was 0.773 (95% confidence interval [CI], 0.594–0.953), which was larger than that of AFP at 0.681 (95% confidence interval [CI], 0.474–0.887). Additionally, the sensitivities of the postoperative MCM6 and AFP were 78.6% and 71.4%, respectively. Notably, the sensitivity of the combination with AFP and MCM6 was increased to 80.1% (Table 4). Harrell C-index could also proved that postoperative serum MCM6 not only significantly correlated with tumor early recurrence but also displayed improved indices of prognostic performance. Harrell C-index = 0.7249 95%CI (0.6143–0.8356). This result showed that the prediction model is accurate and the credibility is high (a C-index of 1 indicates 100% predictive accuracy). To summarize, in patients with resectable HCC, high postoperative serum MCM6 individuals had a higher tendency of early recurrence. In particular, patients with dual positivity of postoperative serum MCM6 and AFP should be supplied with more intensive care and close follow-up after they undergo tumor resection.

**Table 4:** Comparative analysis of early recurrence predictive value of MCM6 and AFP

parameters	AUC (95%CI)	Cut-off value	Sensitivity	Specificity
Preoperative MCM6	0.513 (0.300–0.726)	941.635 (pg/ml)	64.3%	58.8%
Postoperative MCM6	0.773 (0.594–0.953)	941.635 (pg/ml)	78.6%	76.5%
Postoperative AFP	0.681 (0.474–0.887)	20.0 (ng/ml)	71.4%	41.2%
Postoperative (MCM6 & AFP)	_	_	80.1%	77.0%

## Discussion

Invasion and metastasis are responsible for the unsatisfactory long-term survival of HCC patients. An early diagnosis and aggressive intervention may offer HCC patients a significant survival benefit. Thus, the identification of new predictive biomarkers of HCC invasion and prognosis is critical. In this study, we provided evidence that MCM6 had the potential to be a novel prognostic biomarker for HCC patients. IHC assays suggested that MCM6 was highly expressed in HCC tissues. Clinical significance analysis indicated that MCM6 was associated with tumor number, liver cirrhosis and early recurrence. Also, HCC patients with increased MCM6 expression had worse over survival and higher cumulative recurrence rates. All of these results suggested that MCM6 might play an important role in HCC development. In addition, depletion of endogenous MCM6 expression in LM3 and SMMC7721 cells suppressed cell growth, migration, and invasion. Mechanical analysis indicated that MCM6 promoted EMT and activated the MEK/ERK pathway. Moreover, we found that high serum MCM6 levels significantly correlated with high early recurrence risk after hepatectomy. All of these results strongly suggest that MCM6 has the potential to be a useful prognostic biomarker for HCC.

Accumulated data show that EMT results in increasing cell migration and invasion in several cancers [22, 23]. Functional experiments revealed that HCC invasion and metastasis were effectively inhibited by MCM6 knockdown. More significantly, we found that MCM6 knockdown increased epithelial marker expression and decreased mesenchymal marker expression. Additionally, the relationship of MCM6 and EMT markers was confirmed in xenografted tissue specimens. Therefore, our results illustrated that MCM6 promoted the EMT process. Previous reports showed that activation of the MEK/ERK signaling pathway contributed to cell growth, invasion, and EMT [24, 25]. We showed that phosphorylation of ERK was inhibited by MCM6 silencing. This result suggests that MCM6 might affect EMT via regulation of the activation of the ERK signaling pathway. Strategies to target this pathway might be developed to inhibit HCC metastasis, although more mechanistic studies are needed.

Our study found that MCM6 was significantly upregulated in HCC tissues and predicted poor prognosis and high recurrence risk. Data from the TCGA confirmed this result. Since liquid biopsy has been widely applied in diagnostic situations and the detection of many cancers [26, 27], serum biomarkers are also recommended to be used in surveillance for HCC early recurrence. Interestingly, our data indicated that the serum MCM6 protein level can predict early recurrence of HCC patients who accepted radical resection. Plasma analyses suggested that postoperative plasma MCM6 levels of early-recurrence patients were significantly higher than those of non-early-recurrence patients. HCC tissues are not always readily obtained during routine follow-up visits, so MCM6 has meaningful potential to be translated and applied to serum detection although further verification based on large sample tests is needed.

## Conclusions

MCM6 functions as a tumor promotor by activating MEK/ERK signaling and subsequently regulates metastasis and EMT in HCC. In addition, MCM6 can be a potential indicator to predict poor prognosis and early recurrence after receiving curative hepatectomy. These efforts to provide personalized surveillance are expected to improve the overall clinical management of HCC.

## Additional file

Additional file 1: Table S1. The primers used in this study. **Table S2.** Correlation of serum MCM6 expression with clinical variables. (DOCX 19 kb)

## Abbreviations

CCK-8: Cell counting kit-8
EdU: 5-ethynyl-2′-deoxyuridine
ELISA: Enzyme-linked immunosorbent assay
EMT: Epithelial-mesenchymal transition
ERK: Extracellular signal regulated kinase
HCC: Hepatocellular carcinoma
IHC: Immunohistochemistry
MCM6: Minichromosome maintenance complex component 6
qRT-PCR: Quantitative reverse-transcription polymerase chain reaction
ROC: Receiver operating characteristic
ShRNA: Small hairpin RNA
TCGA: The Cancer Genome Atlas
