# Supplementary material for: MCM6 promotes metastasis of hepatocellular carcinoma via MEK/ERK pathway and serves as a novel serum biomarker for early recurrence
Source: J Exp Clin Cancer Res. 2018 Jan 22;37:10. doi: 10.1186/s13046-017-0669-z (PMC5778693; doi:10.1186/s13046-017-0669-z)
Supplement: Additional file 1: Table S1. — The primers used in this study. Table S2. Correlation of serum MCM6 expression with clinical variables. (DOCX 19 kb) [file 13046_2017_669_MOESM1_ESM.docx]

**Supplementary Table 1.** The primers used in this study

| Primers name |  | Sequence (5’-3’) |
| --- | --- | --- |
| MCM6 | Forward | TGTCAGTGGTGTTGATGGATATG |
|  | Reverse | GCTGTCTGTTCCTCATCTCTG |
| E-cadherin | Forward | TCGACACCCGATTCAAAGTGG |
|  | Reverse | TTCCAGAAACGGAGGCCTGAT |
| Vimentin | Forward | TGGCCGACGCCATCAACACC |
|  | Reverse | CACCTCGACGCGGGCTTTGT |
| N-cadherin | Forward | GCGCGTGAAGGTTTGCCAGTG |
|  | Reverse | CCGGCGTTTCATCCATACCACAA |
| Snail | Forward | AAGGATCTCCAGGCTCGAAAG |
|  | Reverse | GCTTCGGATGTGCATCTTGA |
| Fibronectin | Forward | GAAGCTCTCTCTCAGACAACCA |
|  | Reverse | GCCCACGGTAACAACCTCTT |
| ZO-1 | Forward | GCAGCTAGCCAGTGTACAGTATAC |
|  | Reverse | GCCTCAGAAATCCAGCTTCACGAA |

**Supplementary Table 2. Correlation of serum MCM6 expression with clinical variables**

| Characteristics | n | POST OP Serum MCM6 | | *P* value |
| --- | --- | --- | --- | --- |
|  |  | low | high |  |
| Age (years) | | | | |
| ≤ 50 | 13 | 9 (69.2%) | 4 (30.8%) | 0.597 |
| > 50 | 18 | 12 (66.7%) | 6 (33.3%) |  |
| PRE OP Serum AFP (µg/l) | | | | |
| ≤ 20 | 5 | 2 (40.0%) | 3 (60.0%) | 0.176 |
| > 20 | 26 | 19 (73.1%) | 7 (26.9%) |  |
| HBsAg | | | | |
| Negative | 11 | 9 (81.8%) | 2 (18.2%) | 0.202 |
| Positive | 20 | 12 (60.0%) | 8 (40.0%) |  |
| Vascular invasion | | | | |
| No | 17 | 12 (70.6%) | 5 (29.4%) | 0.503 |
| Yes | 14 | 9 (64.3%) | 5 (35.7%) |  |
| Liver cirrhosis | | | | |
| No | 15 | 11 (73.3%) | 4 (26.7%) | 0.398 |
| Yes | 16 | 10 (62.5%) | 6 (37.5%) |  |
| Tumor size (cm) | | | | |
| ≤ 5 | 19 | 12 (63.2%) | 7 (36.8%) | 0.398 |
| > 5 | 12 | 9 (75.0%) | 3 (25.0%) |  |
| Early recurrence | | | | |
| No | 17 | 15 (88.2%) | 2 (11.8%) | **0.001** |
| Yes | 14 | 6 (42.9%) | 8 (57.1%) |  |

**PRE OP: Preoperative, POST OP: Postoperative**
